# A modified melanoma-molGPA scoring model: assessment of survival after and efficacy of different radiotherapy modalities in patients with melanoma brain metastases

**DOI:** 10.1007/s12672-023-00722-2

**Published:** 2023-06-29

**Authors:** Qian Wu, Xueqing Zhang, Hui Li, Lirui Tang, Yibin Zeng, Jing Lin, Yu Chen, Jinluan Li

**Affiliations:** 1 Department of Radiation Oncology, Clinical Oncology School of Fujian Medical University, Fujian Cancer Hospital, Fuzhou, China; 2 Department of Medical Oncology, Clinical Oncology School of Fujian Medical University, Fujian Cancer Hospital, Fuzhou, China

**Keywords:** Brain metastases, Melanoma-molGPA, Metastatic melanoma, Prognostic score, Whole-brain radiotherapy

## Abstract

**Purpose:**

Patients with malignant melanoma brain metastases (MBMs) have poor prognoses. For MBMs, the Melanoma-molGPA is the most widely used predictive score, but its predictive value remains uncertain in patients fully treated with radiotherapy. We identified MBMs prognostic factors and modified the prognostic scoring model.

**Methods:**

We retrospectively analyzed patients diagnosed with MBMs between December 2010 and November 2021 for prognostic factors influencing overall survival (OS) by univariate and multivariate analyses. Nomogram plots were based on Cox regression modeling. We evaluated overall survival (OS) using Kaplan–Meier survival curves and log-rank tests.

**Results:**

The median OS (mOS) was 7.9 months. On multivariate analysis, *BRAF* mutation status (*p* < 0.001), number of brain metastases (BM) (*p* < 0.001), presence of liver metastases (*p* < 0.001), brain metastases with a midline shift (*p* = 0.003), Karnofsky Performance Score (*p* = 0.02), and lymphocyte-to-monocyte ratio (*p* < 0.0001) were independent OS predictors. These were incorporated into a modified risk-stratification model. Overall, whole-brain radiotherapy (WBRT) did not significantly affect mOS (mOS, 6.89 vs. 8.83 months; *p* = 0.07). After risk stratification using our model, WBRT resulted in no significant survival benefit in the low-risk group (mOS 10.07 vs. 13.1 months; *p* = 0.71) but significantly worse prognosis in the high-risk group (mOS, 2.37 vs. 6.92 months; *p* = 0.026).

**Conclusion:**

We propose a modified model that accurately distinguishes the prognosis of patients with MBMs and guides decision-making for radiotherapy. Based on this novel model, WBRT should be cautiously selected for high-risk patients.

**Supplementary Information:**

The online version contains supplementary material available at 10.1007/s12672-023-00722-2.

## Introduction

It is estimated that, by 2022, 97,920 new cases of melanoma will be diagnosed in the USA [1]. The median overall survival (mOS) of melanoma brain metastases (MBMs) is approximately 4–5 months. MBMs arise in 20–54% of patients with advanced melanoma [2, 3]. The prognosis for individuals with MBMs has improved markedly with the continuing development of melanoma treatments, including surgical resection, radiation, and targeted and immunotherapy, either alone or in combination [4, 5].

The blood–brain barrier makes drug therapy for brain tumors difficult; consequently, radiotherapy is a crucial alternative [6]. Whole-brain radiotherapy (WBRT) is essential for the treatment of MBMs; however, its efficacy remains controversial. Some studies have reported that WBRT extends the mOS from 1–2 months to 3–6 months, as compared to supportive treatment. As a complement to neurosurgery, WBRT results in a decreased incidence of intracranial failure and postoperative local recurrence [7]. Another randomized clinical study found that patients who received WBRT plus stereotactic radiosurgery (SRS) died of neurological reasons more often than did those who received SRS alone; thus, the combined treatment offered no survival benefit [8]. Hence, further research on the benefits and drawbacks of WBRT for the treatment of patients with MBMs is essential.

A widely used scoring system called the Melanoma Molecular-Graded Prognostic Assessment (Melanoma-molGPA) was developed by Sperduto et al. Age, Karnofsky Performance Score (KPS), number of brain metastases, number of extracranial metastases, and *BRAF* mutation status were included in the score [9]. However, the effect of applying this score to all patients who received radiotherapy for MBMs with brain metastases remains unclear. Additionally, the score does not consider whether and what type of systemic therapy the patients received. Moreover, few studies have evaluated the efficacy of WBRT using this scoring model.

Therefore, we aimed to modify the Melanoma-molGPA by applying it to patients with MBMs who had undergone brain radiotherapy. Additionally, we used the modified score to explore the effects of different radiotherapy strategies on OS of patients with MBMs.

## Materials and methods

### Patient selection

We investigated 919 patients diagnosed with malignant melanoma at Fujian Cancer Hospital between December 2010 and November 2021. Cases in which no brain metastases were found or documented were excluded. One patient who appeared to have a second primary tumor was also excluded. Cases in which no brain metastases were found or documented, a patient who appeared to have a second primary tumor, and six individuals with insufficient clinical information, were excluded from the study. Sixteen patients who did not receive radiation for the brain metastases were not included, and six patients were disqualified because they had either died or discontinued their treatment prematurely.

This study was approved by the Ethics Committee of the Fujian Cancer Hospital, Fuzhou, China (No. K2023-030-01).

### Variables

The collected information can be divided into three categories. The basic patient information included age, sex, Karnofsky performance status (KPS), time of initial diagnosis, and detection of brain metastases. Primary tumor site, number and maximum diameter of brain metastases, extracranial metastases, midline metastases and synchronous liver metastases, clinical symptoms, *BRAF* mutation status, lymphocyte-to-monocyte ratio (LMR), and serum lactate dehydrogenase (LDH) levels were all included in the tumor information. Treatment information, such as radiotherapy modalities, surgical resection, systemic regimen selection, and regimen cycle were included in the treatment information.

### Statistical analysis

**Fig. 1:**
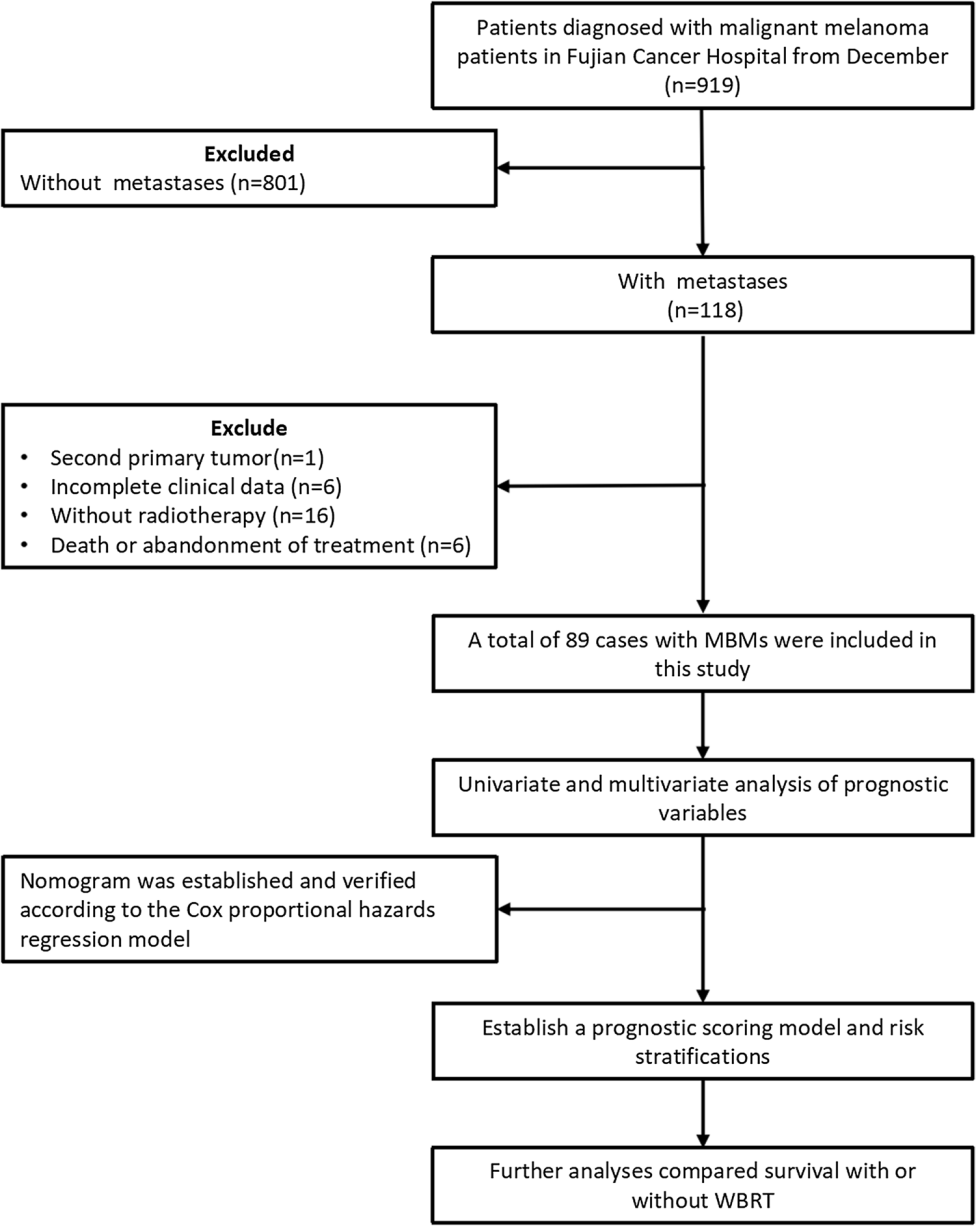
Flowchart of the study design. Abbreviations: MBMs, melanoma brain metastases

The study flow is shown in Fig. 1. The primary endpoint was OS, defined as the time from the first day of diagnosis of brain metastases to death or last follow-up. Univariate and multivariate analyses were performed using Cox proportional hazards regression models to identify the independent prognostic factors. Second, the nomogram of OS rates at 3, 6, and 12 months was plotted based on multivariate analysis results. Harrell’s C-index was used to assess the predictive ability of the model. To verify the accuracy of the nomogram, 1000 iterations of bootstrap resampling iterations were performed.

According to the results of the multivariate analyses, risk scores were derived using the beta regression coefficient. We assumed that the relative risk was 0.5 points for variables > 1 and ≤ 1.5; 1 point for variables > 1.5 but ≤ 2.5; 2 points for variables > 2.5 and ≤ 4; 3 points for variables > 4 and ≤ 6; and 4 points for variables > 6 [10].

Finally, using the cut-off values, the modified scoring model was stratified into low-and high-risk groups. Based on a modified scoring model, survival analysis was performed to determine the effect of WBRT on patients according to their risk. Kaplan–Meier and log-rank tests were used to compare survival differences and to plot survival curves.

Statistical analyses were performed via the package SPSS v.25.0 (IBM Corp., Armonk, NY, USA). The optimal cut-off values of variables were assessed by X-tile software v.3.6.1 (https://x-tile.software.informer.com/3.6/). Nomogram through the "rms" installation package of R software v.4.2.2 (https://www.r-project.org/). *P* values < 0.05 were considered statistically significant.

## Results

### Patient characteristics

Eighty-nine patients (47 men, 42 women) were included in this study. The characteristics of the MBMs are detailed in Additional file 1: Table S1. All patients underwent radiotherapy for brain metastases. The median OS (mOS) was 7.9 months. The proportion of patients with a KPS score of > 80 was 73%. Thirty-three (37%) patients had brain metastases with a maximum diameter ≥ 4 cm, while 56 (63%) had brain metastases < 4 cm. Of the patients, 61.8% had extracranial metastases, and 31% had hemorrhages in the MBMs. Most patients had clinical symptoms (63 patients, 70.8%). WBRT was used for 64 patients (71.9%); 25 (28.1%) were treated with local radiotherapy (LR). Forty (44.9%) patients harbored *BRAF* mutations, while 49 (55.1%) patients harbored wild-type *BRAF*.

### Cox analysis and nomogram establishment

Table 1 shows the results of the univariate and multivariate Cox regression analyses. Univariate analysis revealed that LDH level (*p* = 0.006), presence of hemorrhage in the MBMs (*p* = 0.007), extracranial metastasis (*p* < 0.001), *BRAF* mutation status (*p* < 0.001), number of brain metastases (p < 0.001), maximum diameter of brain metastases (*p* = 0.038), clinical symptoms (*p* = 0.001), KPS (*p* < 0.001), LMR (*p* = 0.001), brain metastases with a midline shift (*p* = 0.003), and the presence of liver metastases (*p* < 0.001) were factors significantly affecting OS (Additional file 2: Figure S1). Among them, *BRAF* mutation status (hazard ratio [HR], 25.415; 95% confidence interval [CI]: 7.830–82.492, *p* < 0.001), number of brain metastases (HR, 2.9; 95%CI 1.493–5.634, *p* = 0.002), KPS (HR, 2.976; 95%CI 1.446–6.126, *p* = 0.003), LMR (HR, 2.579; 95%CI 1.273–5.298, *p* = 0.009), brain metastases with a midline shift (HR, 2.979; 95%CI 1.269–6.994, *p* = 0.002), and the presence of liver metastases (HR, 4.354; 95%CI 2.074–9.137, *p* = 0.002) were independent prognostic factors for OS.

**Table 1:** Univariable and multivariable analyses of covariables associated with OS

Variable	Univariable analysis		Multivariable analysis	
	HR (95% CI)	P	HR (95%CI)	P
Age				
< 70/ ≥ 70	1.035 (0.517–2.074)	0.922		
Sex				
Female/male	0.658 (0.425–1.019)	0.061		
Primary site				
Skin/unknown	0.927 (0.366–2.348)	0.873		
Limb/unknown	1.419 (0.586–3.433)	0.438		
Mucosa/unknown	0.917 (0.374–2.250)	0.860		
Conjunctiva/unknown	1.118 (0.273–4.574)	0.873		
Serum LDH(U/L)				
> ULN/ ≤ ULN	1.89 (41.206–2.973)	0.006	1.672 (0.992- 2.819)	0.053
Presence of hemorrhage in BM				
Yes/no	0.515 (0.317–0.837)	0.007	1.038 (0.533–2.023)	0.913
Extracranial metastases				
Yes/no	4.493 (2.746–8.019)	< 0.001	1.563 (0.766 -3.188)	0.220
BRAF status				
Wild type/mutated	27.034 (10.171–71.860)	< 0.001	25.415 (7.830–82.492)	< 0.001
Number of BM				
≥ 4/ < 4	2.428 (1.483–3.975)	< 0.001	2.9 (1.493–5.634)	0.002
Clinical syndromes				
Yes/no	2.374 (1.423–3.959)	0.001	1.039 (0.546–1.977)	0.907
KPS (%)				
< 80/ ≥ 80	4.918 (2.956–8.182)	< 0.001	2.976 (1.446–6.126)	0.003
Maximum diameter of BM (cm)				
≥ 4/ < 4	1.632 (1.029–2.590)	0.038	1.352 (0.748–2.445)	0.318
BM with a midline shift				
Yes/no	3.453 (1.694–7.037)	0.001	2.979 (1.269–6.994)	0.012
Presence of liver metastases				
Yes/no	6.564 (3.87–11.118)	< 0.001	4.354 (2.074–9.137)	< 0.001
LMR				
< 2/ ≥ 2	3.564 (2.154–5.897)	< 0.001	2.597 (1.273–5.298)	0.009

*BM* brain metastasis, *KPS* Karnofsky performance status, *LMR* lymphocyte-to-monocyte ratio, *LDH* lactate dehydrogenase, *ULN* upper limit of normal, BRAF status, Serine/threonine protein kinase, encoded on chromosome 7q34, that activates the MAP kinase/ERK-signaling pathway

**Fig. 2:**
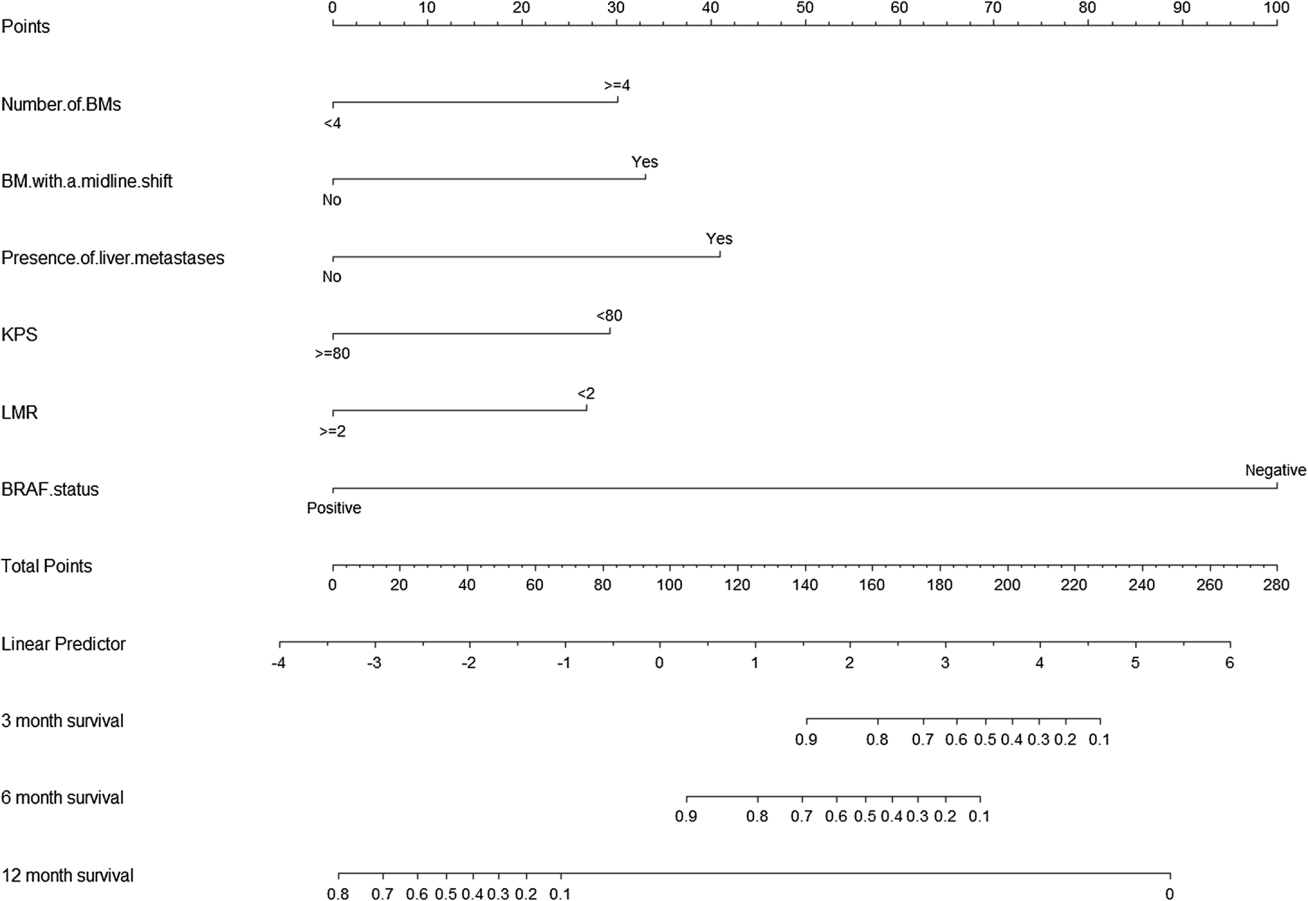
Nomogram predicting the overall survival (OS) rates at 3, 6 and 12 months for patients with melanoma brain metastases. The nomogram summed the points identified on the scale for each variable. The total points projected on the bottom scales indicate the probabilities of OS rates at 3, 6 and 12 months. Abbreviations: BM, brain metastasis; KPS, Karnofsky performance status; LMR, lymphocyte-to-monocyte ratio; BRAF status, Serine/threonine protein kinase, encoded on chromosome 7q34, that activates the MAP kinase/ERK-signaling pathway

The nomograms of 3 month, 6 month, and 12 month OS were plotted based on the analysis results of the Cox regression model (Fig. 2). The C-index, used to represent the accuracy of nomogram, was 0.866 (95%CI 0.824–0.912).

### Development of modified scoring models

Based on the combined results of Cox and Melanoma-molGPA analyses, we developed a modified scoring model for better prediction of the prognosis of patients with MBMs who received radiotherapy. The risk factors and point allocation in the modified Melanoma-molGPA were as follows (Table 2): The presence of hemorrhage in the MBM, clinical syndromes (yes) in the MBMs, or maximum diameter of brain metastases (BM) (≥ 4): 0.5 points each. Serum LDH > upper limits of normal (ULN), or the presence of extracranial metastases: 1 point each. KPS < 80, LMR < 2, the presence of a midline shift of the MBMs, or number of MBMs ≥ 4: 2 points, each. The presence of liver metastases: 3 points. Wild-type *BRAF* status: 4 points. The C-index of the modified Melanoma-molGPA was 0.886 (95%CI 0.871–0.924). The total score range was 0–18.5 points, and the optimal cutoff point was 7.75. Patients were divided into two risk groups based on the cutoff score: 52 (58.4%) patients were assigned to the low-risk group (score < 7.75) and 37 (41.6%) were assigned to the high-risk group (score ≥ 7.75).

**Table 2:** Risk variables for the scoring system

Risk variable	Exp(B)	Risk score
Serum LDH(U/L)	1.672	
> ULN		1
≤ ULN		0
Presence of hemorrhage in BM	1.038	
Yes		0.5
No		0
Extracranial metastases	1.563	
Yes		1
No		0
BRAF status	25.415	
Wild type		4
Mutated		0
Number of BM	2.900	
≥ 4		2
< 4		0
Clinical Syndromes	1.039	
Yes		0.5
No		0
KPS (%)	2.976	
< 80		2
≥ 80		0
Maximum diameter of BM (cm)	1.352	
≥ 4		0.5
< 4		0
LMR	2.579	
< 2		2
≥ 2		0
Presence of liver metastases	4.354	
Yes		3
No		0
BM with a midline shift	2.979	
Yes		2
No		0

BM brain metastasis, *LDH* lactate dehydrogenase, *ULN* upper limit of normal, *BRAF *status, Serine/threonine protein kinase, encoded on chromosome 7q34, that activates the MAP kinase/ERK-signaling pathway; *KPS* Karnofsky performance status, *LMR* lymphocyte-to-monocyte ratio

### Comparison of prognosis based on risk stratification

**Fig. 3:**
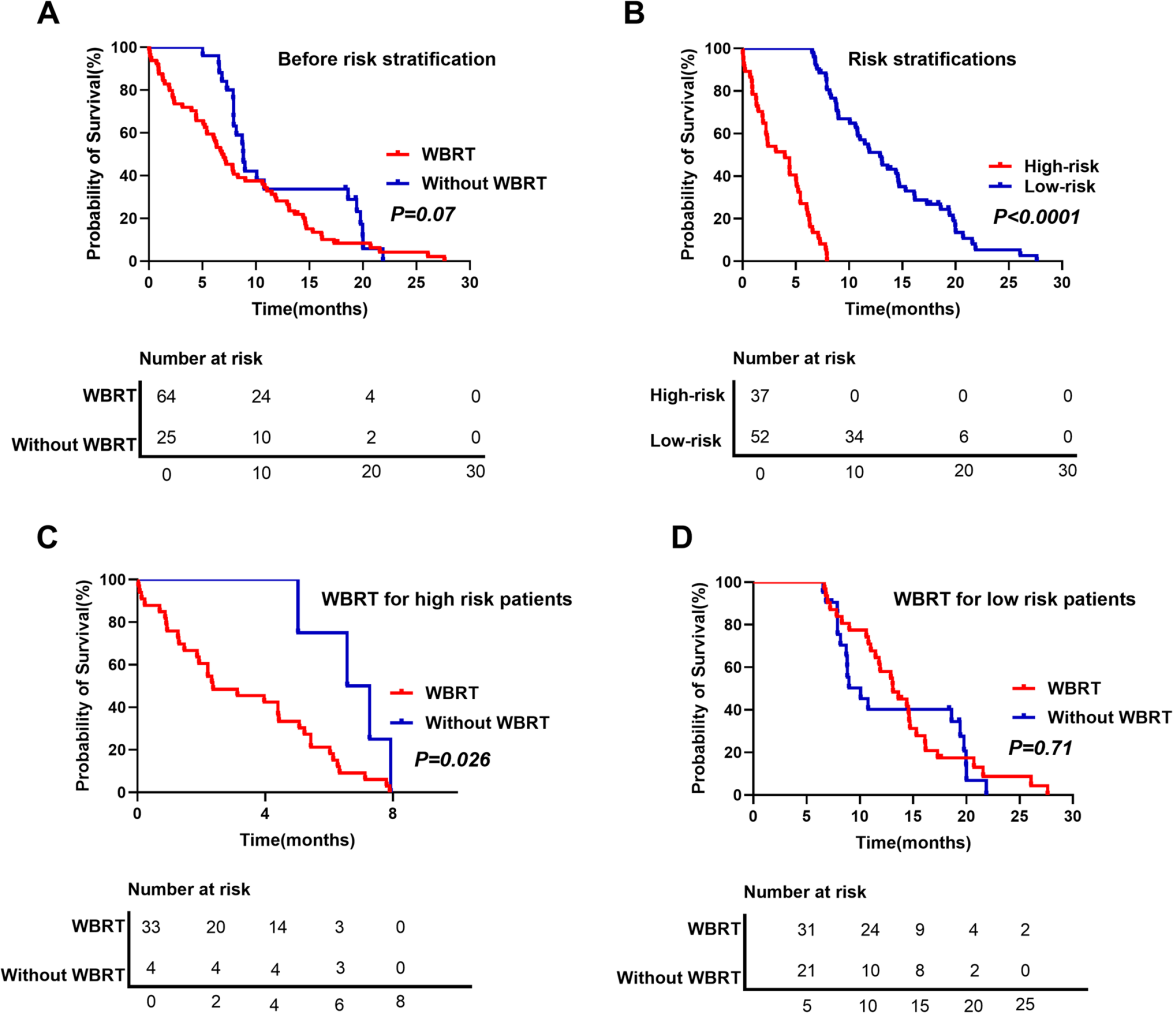
**A** Comparison of overall survival (OS) between the whole-brain radiotherapy (WBRT) and without WBRT groups. **B** Kaplan–Meier curves stratified based on the risk stratification in the high-risk group (3.75–13 points) and low-risk group (< 3.75 points) **C** OS of patients in the high-risk group stratified according to WBRT and without WBRT radiotherapy **D** OS of patients in the low-risk group stratified according to WBRT and without WBRT radiotherapy

Before dividing the patients based on stratification by modified Melanoma-molGPA score, we found no difference in OS between patients who did and who did not undergo WBRT (mOS, 6.89 vs. 8.83 months; *p* = 0.07, Fig. 3A). However, with risk stratification, the overall OS was significantly better in the low-risk group than in the high-risk group (mOS, 12.9 vs. 3.97 months; *p* < 0.001, Fig. 3B). Furthermore, we found no evidence of a survival benefit from WBRT in low-risk patients (mOS, 10.07 vs. 13.1 months; *p* = 0. 71, Fig. 3C). In addition, we found that high-risk MBM patients who had undergone WBRT had a worse mOS than that of those who did not undergo WBRT (mOS, 6.92 vs. 2.37 months; *p* = 0.026, Fig. 3D).

## Discussion

Melanomas are biologically heterogeneous, making it difficult to predict the prognosis of patients with MBMs[11]. In this study, we successfully constructed a novel scoring model and analyzed the efficacy of radiotherapy modalities. We showed that WBRT was harmful to patients stratified as high-risk (*p* = 0.026) based on the novel Melanoma-molGPA score.

In our scoring model, the presence of liver metastases, LMR, and MBM with a midline shift were newly added to the modified Melanoma-molGPA as independent prognostic factors. A recent study reported that patients with stage IV melanoma presenting with liver metastases tended to have worse survival rates than those with lung metastases (*p* < 0.0001) [12]. Another study involving 357 patients with melanoma found that liver metastases were associated with worse survival outcomes (*p* = 0.004) [13]. Similarly, in our study, Cox regression analysis identified the presence of liver metastases as an independent prognostic factor affecting OS in patients with MBMs (HR, 4.354; *p* = 0.002). Furthermore, melanoma patients who present with liver metastases had worse OS than those without MBMs (*p* < 0.0001). The mechanism underlying the lower survival rate of patients with melanoma with liver metastases remains to be investigated. Chattopadhyay et al. reported that this was associated with liver growth factors (IGF), and that inhibiting the IGF-1 signaling pathway could achieve a therapeutic effect by inhibiting tumor growth [14]. LMR is an inflammatory marker that has been shown to have prognostic value in various types of tumors [15]. A study enrolling 156 patients with esophageal cancer demonstrated that a low LMR was an independent prognostic factor for poor survival (HR, 2.56; *p* = 0.03) [16]. Similarly, our findings showed that the LMR was an independent prognostic factor affecting OS in patients with MBMs (HR, 2.579; *p* = 0.009). The high LMR group had better OS outcomes than did the low LMR group (*p* = 0.0001). Deacu et al. found that a midline shift in brain metastases was an independent risk factor for mortality (HR, 1.15; p = 0.025) [17]. Similar findings were observed in our study, where we found that the presence of brain metastases with midline shifts were an independent prognostic factor affecting OS (HR, 2.979;* p* = 0.002) and were associated with a worse prognosis (*p* = 0.003).

In our patient cohort, the number of patients with *BRAF* mutations (n = 40) and wild-type *BRAF* (n = 49) was similar, with the mutant type accounting for 44.9% of patients. According to the beta values of the Cox regression model in this study, *BRAF* status was the strongest prognostic factor (HR, 25.415; *p* < 0.001). This was consistent with previous findings [18, 19]. A retrospective study by Frinton et al. reported that patients with *BRAF* mutations had significantly better survival than did those with wild-type *BRAF* (*p* = 0.0039) [20]. Consistently, we also found that patients with *BRAF* mutations tended to have longer survival (*p* < 0.001). This may be due to the benefits of the BRAF inhibitor therapy.

WBRT is an important tool for the local treatment of MBMs; however, its survival benefits are debated. It has been suggested that, in the case of stable extracranial disease or as an adjunct to SRS or neurosurgery, WBRT can control the progression of intracranial disease [21]. However, a phase 3 clinical trial found that adjuvant WBRT for 1–3 MBMs provided no clinical benefit in terms of distant intracranial control, survival, or improved performance status [22]. In this study, there was no significant difference in OS between the patients overall (without risk stratification) with or without WBRT (*p* = 0.07). To determine a more appropriate prognostic score for patients with MBMs in the radiotherapy population, we performed risk stratification using our novel modified scoring model. Our study showed no statistically significant differences in the survival of individuals in the low-risk group with or without WBRT (*p* = 0.71). Nevertheless, importantly, in the high-risk group, patients who underwent WBRT had worse OS outcomes than those who did not (*p* = 0.026). Thus, according to our research, WBRT may not offer a survival benefit for patients with MBMs, particularly for those with a heavy tumor burden and who have more risk factors. In this study, a certain proportion of patients presented with extracranial metastases, and the presence of hemorrhage in the MBM as a neurological emergency cannot be ignored (61% and 31%, respectively). Moreover, WBRT is often used as a palliative measure for patients who have exhausted treatment for multiple disseminated lesions, resulting in selection bias in the patient population [20]. In addition, we suggest that before selecting WBRT, the potential side-effects on neurocognitive function must be considered.

This study also had some limitations. First, it was a single-center retrospective study that lacked accessment of the quality of life. Second, the reasons underlying the worse survival outcomes of high-risk patients need to be explored further. Third, and similar to other studies, the number of patients included in this study was limited due to the low incidence of melanoma. This limits our findings in terms of guiding decision-making regarding radiotherapy.

## Conclusion

In summary, a modified Melanoma-molGPA was successfully established to evaluate the efficacy of different radiotherapy strategies in melanoma brain metastases. Analysis of treatment decisions revealed that patients with high-risk factors receiving WBRT should be cautious.

## Supplementary Information

**Additional file 1: TableS1.** Patient baseline characteristics

**Additional file 2: Fig.S1.** Kaplan–Meier survival curves showing the overall survival.

## Data Availability

The datasets generated during and/or analysed during the current study are available from the corresponding author on reasonable request.

## References

[1] Siegel RL, Miller KD, Fuchs HE, Jemal A (2022). Cancer statistics, 2022. CA Cancer J Clin.

[2] Gibney GT, Forsyth PA, Sondak VK (2012). Melanoma in the brain: biology and therapeutic options. Melanoma Res.

[3] Franceschini D, Franzese C, Navarria P, Ascolese AM, De Rose F, Del Vecchio M (2016). Radiotherapy and immunotherapy: can this combination change the prognosis of patients with melanoma brain metastases?. Cancer Treat Rev.

[4] Gaudy-Marqueste C, Dussouil AS, Carron R, Troin L, Malissen N, Loundou A (2017). Survival of melanoma patients treated with targeted therapy and immunotherapy after systematic upfront control of brain metastases by radiosurgery. Eur J Cancer.

[5] White RJ, Abel S, Horne ZD, Lee J, Edington H, Greenberg L (2020). Melanoma brain metastases: Is it time to eliminate radiotherapy?. J Neurooncol.

[6] Ghasempour E, Hesami S, Movahed E, Keshel SH, Doroudian M (2022). Mesenchymal stem cell-derived exosomes as a new therapeutic strategy in the brain tumors. Stem Cell Res Ther.

[7] Jiang C, Wallington DG, Anker CJ, Lawson DH, Yushak ML, Kudchadkar RR (2020). Changing therapeutic landscape for melanoma with multiple brain metastases. Neurosurgery.

[8] Aoyama H, Shirato H, Tago M, Nakagawa K, Toyoda T, Hatano K (2006). Stereotactic radiosurgery plus whole-brain radiation therapy vs stereotactic radiosurgery alone for treatment of brain metastases: a randomized controlled trial. JAMA.

[9] Sperduto PW, Jiang W, Brown PD, Braunstein S, Sneed P, Wattson DA (2017). Estimating survival in melanoma patients with brain metastases: an update of the graded prognostic assessment for melanoma using molecular markers (melanoma-molGPA). Int J Radiat Oncol Biol Phys.

[10] Guédon A, Blauwblomme T, Boulouis G, Jousset C, Meyer P, Kossorotoff M (2018). Predictors of outcome in patients with pediatric intracerebral hemorrhage: development and validation of a modified score. Radiology.

[11] Zhang X, Tai Z, Miao F, Huang H, Zhu Q, Bao L (2022). Metabolism heterogeneity in melanoma fuels deactivation of immunotherapy: predict before protect. Front Oncol.

[12] Conway JW, Rawson RV, Lo S, Ahmed T, Vergara IA, Gide TN (2022). Unveiling the tumor immune microenvironment of organ-specific melanoma metastatic sites. J Immunother Cancer.

[13] Waninger JJ, Ma VT, Journey S, Skvarce J, Chopra Z, Tezel A et al. 2021. Validation of the American Joint Staging of Patients With Metastatic Cutaneous Melanoma Treated With Immune Checkpoint Inhibitors. JAMA Netw Open. 10.1001/jamanetworkopen.2021.0980 10.1001/jamanetworkopen.2021.0980 PMC7944385 33687443

[14] Chattopadhyay C, Bhattacharya R, Roszik J, Khan FS, Wells GA, Villanueva H (2022). Targeting IRS-1/2 in uveal melanoma inhibits in vitro cell growth, survival and migration, and in vivo tumor growth. Cancers.

[15] Dotto-Vasquez G, Villacorta-Ampuero AK, Ulloque-Badaracco JR, Hernandez-Bustamante EA, Alarcón-Braga EA, Herrera-Añazco P (2022). Lymphocyte-to-monocyte ratio and clinical outcomes in cholangiocarcinoma: a systematic review and meta-analysis. Diagnostics.

[16] Chen CJ, Lee CT, Tsai YN, Tseng CM, Chen TH, Hsu MH (2022). Prognostic significance of systemic inflammatory response markers in patients with superficial esophageal squamous cell carcinomas. Sci Rep.

[17] Deacu M, Popescu S, Docu Axelerad A, Topliceanu TS, Aschie M, Bosoteanu M (2022). Prognostic factors of low-grade gliomas in adults. Curr Oncol.

[18] Ramanujam S, Schadendorf D, Long GV (2015). Systemic therapies for melanoma brain metastases: Which drug for whom and when?. Chin Clin Oncol.

[19] Davies MA, Liu P, McIntyre S, Kim KB, Papadopoulos N, Hwu WJ (2011). Prognostic factors for survival in melanoma patients with brain metastases. Cancer.

[20] Frinton E, Tong D, Tan J, Read G, Kumar V, Kennedy S (2017). Metastatic melanoma: prognostic factors and survival in patients with brain metastases. J Neurooncol.

[21] Dyer MA, Arvold ND, Chen YH, Pinnell NE, Mitin T, Lee EQ (2014). The role of whole brain radiation therapy in the management of melanoma brain metastases. Radiat Oncol.

[22] Hong AM, Fogarty GB, Dolven-Jacobsen K, Burmeister BH, Lo SN, Haydu LE (2019). Adjuvant whole-brain radiation therapy compared with observation after local treatment of melanoma brain metastases: a multicenter, randomized phase III trial. J Clin Oncol.

