# Supplementary material for: A modified melanoma-molGPA scoring model: assessment of survival after and efficacy of different radiotherapy modalities in patients with melanoma brain metastases
Source: Discov Oncol. 2023 Jun 29;14:116. doi: 10.1007/s12672-023-00722-2 (PMC10310639; doi:10.1007/s12672-023-00722-2)
Supplement: Supplementary file 1 — Additional file 1: TableS1. Patient baseline characteristics [file 12672_2023_722_MOESM1_ESM.docx]

Additional file material 1

**TableS1** Patient baseline characteristics

| Variable | Number of patients (n=89, %) |
| --- | --- |
| Age |  |
| ≥70 | 9(10.1) |
| <70 | 80(89.9) |
| Sex |  |
| Male | 47(52.8) |
| Female | 42(58.4) |
| Primary site |  |
| Skin | 21(23.6) |
| Limb | 30(33.7) |
| Mucosa | 28(31.5) |
| Conjunctiva | 3(3.3) |
| Unknown | 7(7.9) |
| KPS (%) |  |
| ≥80 | 65(73.0) |
| <80 | 24(27.0) |
| Serum LDH (U/L) |  |
| ≤ULN | 36(40.4) |
| >ULN | 53(59.6) |
| LMR |  |
| ≥2 | 64(72) |
| <2 | 25(28) |
| BM with a midline shift |  |
| Yes | 9(10.0) |
| No | 80(90.0) |
| Number of BM |  |
| ≥4 | 25 (28.0) |
| <4 | 64(72.0) |
| Maximum diameter of BM (cm) |  |
| <4 | 56(63.0) |
| ≥4 | 33(37.0) |
| Extracranial metastases |  |
| Yes | 55(61.8) |
| No | 34(38.2) |
| Presence of liver metastases |  |
| Yes | 32(36) |
| No | 57(64) |
| Clinical Syndromes |  |
| Yes | 63(70.8) |
| No | 26(29.2) |
| Presence of hemorrhage in BM |  |
| Yes | 28 (31) |
| No | 61(69) |
| Radiotherapy strategies |  |
| LR | 25(28.1) |
| WBRT + LR | 26(29.2) |
| WBRT | 38(42.7) |
| Concomitant systemic therapy (receiving at least 1 cycle, at any given time) |  |
| IT/TT/IT+TT | 59(66.3) |
| Only chemotherapy | 16(20) |
| None | 14(15.7) |
| BRAF status |  |
| Mutated | 40(44.9) |
| Wild type | 49(55.1) |

Abbreviations: BM, brain metastasis；KPS, Karnofsky performance status; LMR, lymphocyte-to-monocyte ratio; LDH，lactate dehydrogenase；ULN, upper limit of normal；BRAF，Serine/threonine protein kinase, encoded on chromosome 7q34, that activates the MAP kinase/ERK-signaling pathway; WBRT, whole-brain radiotherapy；LR,local radiotherapy; IT，immunotherapy, TT, targeted therapy.
