# Supplementary material for: A modified melanoma-molGPA scoring model: assessment of survival after and efficacy of different radiotherapy modalities in patients with melanoma brain metastases
Source: Discov Oncol. 2023 Jun 29;14:116. doi: 10.1007/s12672-023-00722-2 (PMC10310639; doi:10.1007/s12672-023-00722-2)
Supplement: Supplementary file 2 — Additional file 2: Fig.S1. Kaplan–Meier survival curves showing the overall survival. [file 12672_2023_722_MOESM2_ESM.docx]

Supplementary material 2


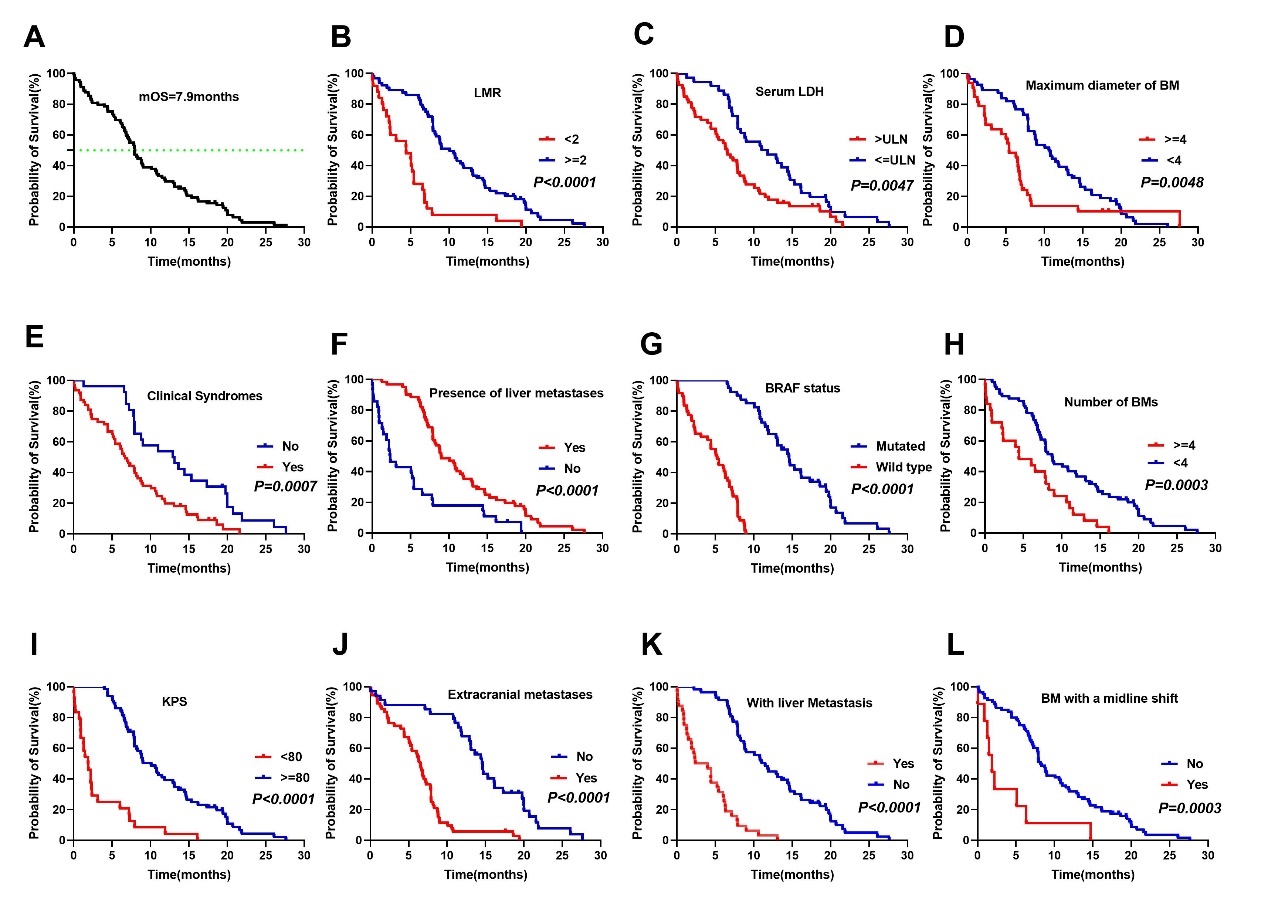


FigureS1: Kaplan–Meier survival curves showing the overall survival

(A) overall survival (OS) of the entire cohort; (B)melanoma brain metastases patients stratified according to lymphocyte-to-monocyte ratio;(C)Serum LDH levels; (D)Maximum diameter of BM;(E)Clinical symptoms;(F) Hemorrhage present in BM;(G)BRAF gene status;(H)Number of brain metastases;(I)Karnofsky performance score;(J)Extracranial metastases;(K) With Liver Metastasis;(L) Median line shift of BM
